# Discriminators of pseudoprogression and true progression in high-grade gliomas: A systematic review and meta-analysis

**DOI:** 10.1038/s41598-022-16726-x

**Published:** 2022-08-02

**Authors:** Chris Taylor, Justyna O. Ekert, Viktoria Sefcikova, Naomi Fersht, George Samandouras

**Affiliations:** 1 UCL Queen Square Institute of Neurology, University College London, Gower St., Bloomsbury, Queen Square, London, WC1E 6BT UK; 2 Wellcome Centre for Human Neuroimaging, University College London, 12 Queen Square, London, UK; 3 Department of Oncology, University College London Hospitals NHS Foundation Trust, London, UK; 4 Victor Horsley Department of Neurosurgery, The National Hospital for Neurology and Neurosurgery, Queen Square, London, UK

**Keywords:** Cancer imaging, Cancer prevention, Tumour biomarkers, Cancer, Biomarkers, Signs and symptoms

## Abstract

High-grade gliomas remain the most common primary brain tumour with limited treatments options and early recurrence rates following adjuvant treatments. However, differentiating true tumour progression (TTP) from treatment-related effects or pseudoprogression (PsP), may critically influence subsequent management options. Structural MRI is routinely employed to evaluate treatment responses, but misdiagnosis of TTP or PsP may lead to continuation of ineffective or premature cessation of effective treatments, respectively. A systematic review and meta-analysis were conducted in accordance with the Preferred Reporting Items for Systematic Reviews and Meta-analyses method. Embase, MEDLINE, Web of Science and Google Scholar were searched for methods applied to differentiate PsP and TTP, and studies were selected using pre-specified eligibility criteria. The sensitivity and specificity of included studies were summarised. Three of the identified methods were compared in a separate subgroup meta-analysis. Thirty studies assessing seven distinct neuroimaging methods in 1372 patients were included in the systematic review. The highest performing methods in the subgroup analysis were DWI (AUC = 0.93 [0.91–0.95]) and DSC-MRI (AUC = 0.93 [0.90–0.95]), compared to DCE-MRI (AUC = 0.90 [0.87–0.93]). 18F-fluoroethyltyrosine PET (18F-FET PET) and amide proton transfer-weighted MRI (APTw-MRI) also showed high diagnostic accuracy, but results were based on few low-powered studies. Both DWI and DSC-MRI performed with high sensitivity and specificity for differentiating PsP from TTP. Considering the technical parameters and feasibility of each identified method, the authors suggested that, at present, DSC-MRI technique holds the most clinical potential.

## Introduction

High-grade gliomas (HGGs) remain some of the most common subtypes of primary brain tumours^1^ with standard treatment options including surgical debulking followed by radiotherapy, and adjuvant or concurrent chemoradiation therapy (CCRT) with temozolomide, in world health organisation (WHO) grade III and IV, respectively^2,3^. However, HGGs are characterised with early and high recurrence rates^4^. Routine MRI scans are often reported to display tumour volume growth, new or enlarged areas of contrast enhancement, or oedema which may represent either TTP or treatment effects called PsP. The latter can be transient and is often clinically asymptomatic^5^, but is often misdiagnosed as TTP, leading to premature cessation of potentially effective treatments and often substitution for less effective, second-line treatments^6^. Conversely, TTP misdiagnosed as PsP can complicate the monitoring of tumour progression by increasing waiting times, negatively influencing treatment outcomes^7^.

A meta-analysis by Abbasi and colleagues^8^ found a form of pseudoprogression in 36% of 2603 patients harbouring HGGs. However, lack of standardised definitions for PsP and accurate diagnostic methods resulted in varying prevalence estimates in the literature. PsP occurs within the first 3 months following radiotherapy almost 60% of the time, with a range that is usually between 2 and 6 months^9,10^. It is physiologically and clinically comparable to radiation necrosis, which generally occurs 3–12 months after therapy^11^. It is postulated that radiotherapy induces local endothelial cell death, leading to increased vascular permeability, perilesional oedema, and mass effect^12^. This gives a localised area of contrast enhancement on structural MRI that requires further assessment to distinguish from TTP^12^. Interestingly, patients with confirmed PsP generally have an improved prognosis^13^, although this may be subject to survivor bias due to the usually longer time PsP requires to manifest^5^.

Standard practice currently involves clinical confirmation of PsP using serial MRI, histopathology via invasive brain biopsy, and/or application of the RANO criteria^14^. This practice is time-consuming and can be subjective, and histological confirmation requires admission and additional surgery under general anaesthetic. Watchful surveillance can further delay clinical decision making, significantly affecting prognosis^7^. Several imaging methods allowing early differentiation between PsP and TTP, are currently under investigation and demonstrate high sensitivity and specificity compared to histological confirmation.

Current work has applied dynamic susceptibility contrast perfusion MRI (DSC-MRI), dynamic contrast-enhanced perfusion MRI (DCE-MRI), diffusion-weighted MRI (DWI), arterial spin labelling (ASL), amide proton transfer-weighted MRI (APTw-MRI), 18F-fluoroethyltyrosine PET (FET-PET), and combinations of these modalities. All considered modalities have shown a degree of diagnostic value, but their relative clinical potential is still not well established. The aim of this systematic review and meta-analysis is to provide a comprehensive comparison of PsP and TTP differentiators based on measures of sensitivity, specificity, and clinical applicability. The advent of novel, more quantitative methods of diagnosis are also discussed.

## Methods

### Inclusion/exclusion criteria

#### Study design

Randomised controlled trials, controlled clinical trials, and prospective or retrospective observational studies were included with a sample size threshold n ≥ 10. Conference abstracts, grey literature, and articles with no available English translation were excluded at the screening stage.

#### Population

Studies included adult patients (≥ 16 years old) receiving radiotherapy or chemoradiotherapy following a diagnosis of a high-grade glioma. Studies needed to specify the proportion of the sample that exhibited PsP, which was confirmed no more than 6 months following radiotherapy.

#### Intervention

Studies assessed the diagnostic accuracy of a method used to differentiate TTP from PsP in HGG patients.

#### Outcome

Study outcomes reported a method’s diagnostic sensitivity and specificity compared to the gold standard of expert assessment, and/or according the RANO criteria.

### Search strategy

**Figure 1:**
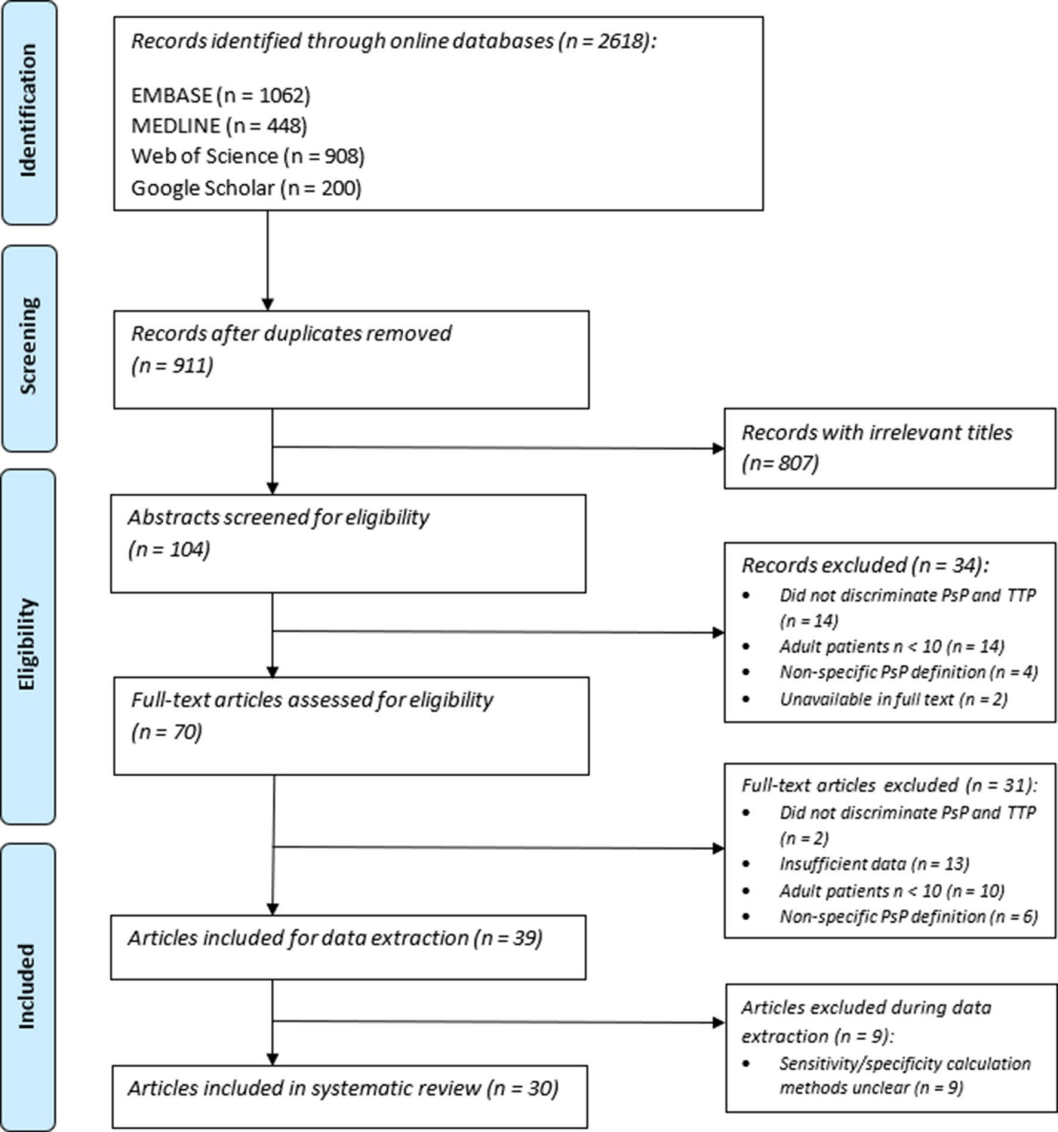
PRISMA flow chart of the study selection process. sP = pseudoprogression; TTP = true tumour progression.

The search strategy was devised in line with the recommendations in Bramer and colleagues^15^. The entirety of Embase, MEDLINE, and Web of Science were searched on the 20th of May 2022. The first 200 results of a Google Scholar search were also included. The full search strategy is detailed in Supplementary Material A. Studies returned by the search were compiled and screened for data extraction in accordance with the Preferred Reporting Items for Systematic Reviews and Meta-Analysis (PRISMA) method^16^. All titles and selected abstracts were screened on the basis of the inclusion criteria, and full texts were subsequently reviewed. The full selection process is shown in Fig. 1, and the full exclusion criteria listed in Supplementary Material C. The protocol was registered to PROSPERO prior to searching (ID CRD42022218217).

### Data extraction and quality assessment

Data were extracted onto a spreadsheet with the following variables: (1) first author and year of publication; (2) neuroimaging method of discrimination between TTP and PsP; (3) sample size and demographics, including proportion of patients that presented with PsP; (4) administration of radiotherapy / chemoradiation therapy / CCRT; (5) tumour grade; and (6) sensitivity and specificity measures. Extracted data were inputted into Cochrane Review Manager 5.4^17^. The review was conducted according to the Meta-analysis of Observational Studies in Epidemiology (MOOSE) proposal^18^. The quality of included studies and risk of bias was assessed with the Quality Assessment of Diagnostic Accuracy (QUADAS-2) tool by two independent and blinded reviewers C.T. and V.S^19^. Any disagreement was resolved with consensus.

In the included studies, sensitivity was defined as the proportion of patients with histopathologically confirmed TTP that presented as such with the modality of choice, or the true positive rate. Specificity was defined as the proportion of patients with histopathologically confirmed PsP that presented as such, or the true negative rate. A high sensitivity and specificity constitute a high diagnostic accuracy, which in turn represents the overall precision of clinical decisions. Studies that did not meet this definition of sensitivity and specificity were excluded during data extraction.

### Data synthesis and statistical analysis

All included studies were presented in a forest plot. A separate subgroup meta-analysis was performed to compare the three most prevalent methods among studies: DSC-MRI (n = 12), DCE-MRI (n = 4), and DWI (n = 12). For the systematic review, the primary outcomes were TTP and PsP discrimination method sensitivity and specificity. For the subgroup meta-analysis, the primary outcomes were pooled sensitivity, specificity, and area under the summary receiver operating characteristics curve (SROC AUC)^20^.

Within-group heterogeneity was assessed using the *I*^*2*^ variable, which describes the proportion of variation in study results that can be attributed to heterogeneity^21^. To account for the high heterogeneity of data across multiple modalities, data inputted into Cochrane Review Manager 5.4 were analysed using a random effects model, which assumes individual effects are uncorrelated with independent variables. Pooled sensitivity, specificity, likelihood ratio, negative likelihood ratio, diagnostic odds ratio (DOR) and SROC AUC were calculated using the MIDAS (meta-analytical integration of diagnostic test accuracy studies) package in STATA^22^. Forest plots and a subgroup analysis SROC plot were generated in Cochrane Review Manager 5.4. For studies reporting median instead of mean age, the mean was estimated according to previously established methods^23^.

### Consent to participate

Informed consent was obtained from all individual participants included in the study.

## Results

### Search results

Following deduplication, the literature search yielded 911 abstracts. Following abstract screening, 70 full-text articles were retrieved and assessed for eligibility. Data was extracted from 39 articles, 9 of which did not report sufficient information to allow for calculation of sensitivity and specificity values, and therefore were excluded. A total of 30 studies totalling 1372 patients were included in the systematic review (see Table 1).

**Table 1:** Details of included studies.

Method	Study	Sample size	PsP/TTP	M/F	Mean age (yrs)	WHO grade	Radiation therapy
DSC-MRI	Baek et al.^63^	79	37/42	46/33	51	IV	CRT-TMZ
	Cha et al.^64^	35	24/11	18/17	49	IV	CRT-TMZ
	Kerkhof et al.^34^	58	26/32	41/17	60	IV	CRT-TMZ
	Kong et al.^65^	59	26/33	49/10	50	IV	CRT-TMZ
	Mangla et al.^66^	19	7/12	NS	63	IV	RT-TMZ
	Martínez-Martínez et al.^37^	34	17/17	14/20	48	III–IV	CRT-TMZ
	Mihailović et al.^67^	40	8/32	37/3	51	IV	RT-TMZ
DSC-MRI, DWI	Kim et al.^24^	34	20/14	25/9	62	IV	CRT-TMZ
	Prager et al.^25^	51	8/43	NS	55	IV	CRT-TMZ
DSC-MRI, ASL	Jovanovic et al. 2017^27^	31	11/20	21/10	49	IV	RT-TMZ
DSC-MRI & ASL	Choi et al.^28^	62	28/34	37/25	49	IV	CRT-TMZ
DSC-MRI & DCE-MRI	Elshafeey et al.^29^	98	22/76	57/41	50	IV	CRT-TMZ
DSC-MRI, DWI, DSC-MRI & DWI	Shi et al.^26^	34	12/22	24/10	47	III-IV	CRT-TMZ
DWI	Bulik et al.^68^	24	6/18	17/7	50	IV	CRT-TMZ
	Chu et al.^69^	30	15/15	16/14	51	IV	CRT-TMZ
	Kazda et al. 2016^70^	39	10/29	28/11	51	IV	CRT-TMZ
	Lee et al.^71^	22	12/10	NS	49	IV	CRT-TMZ
	Patel et al.^72^	76	30/46	46/30	56	IV	CRT-TMZ
	Reimer et al.^73^	35	7/28	26/9	58	III-IV	CRT-TMZ
	Song et al.^74^	20	10/10	10/10	51	IV	CRT-TMZ
	Wu et al.^75^	40	16/24	28/12	46	III/IV	CRT-TMZ
	Yoo et al.^76^	42	18/24	27/15	61	IV	CRT-TMZ
DCE-MRI	Nam et al.^77^	37	22/15	26/11	58	IV	CRT-TMZ
	Suh et al.^78^	79	37/42	36/43	49	IV	CRT
	Thomas et al.^79^	37	13/24	25/12	63	IV	CRT-TMZ
	Yun et al.^80^	33	16/17	22/11	55	IV	CRT-TMZ
18F-FET PET	Galldiks et al.^30^	22	11/11	14/8	56	IV	CRT-TMZ
APTw-MRI	Ma et al.^31^	32	12/20	21/11	56	III-IV	CRT-TMZ
Conventional MRI*	Sun et al.^81^	77	26/51	40/37	49	IV	CCRT-TMZ
	Young et al.^32^	93	30/63	58/35	59	IV	CRT-TMZ

APTw-MRI = amide proton transfer-weighted MRI; ASL = arterial spin labelling; CRT-TMZ = chemoradiotherapy with adjuvant temozolomide; NS = not specified, TTP = true progression. Conventional MRI included contrast-enhanced T1-weighted and T2-weighted acquisitions.

Of the 1372 patients, 538 (39.2%) cases were confirmed to have PsP following radiotherapy, according to histological examination and/or examination according the RANO criteria. The mean age across the studies was 54, with a range of study mean age of 46 to 62 years. Based on 27 studies with relevant reporting, the male/female ratio was 1.4/1.

Seven distinct methods for differentiating PsP from TTP were identified: DSC-MRI, DWI, DCE-MRI, ASL, APTw-MRI, 18F-FET PET, and conventional MRI. Three studies compared DSC-MRI and DWI^24–26^ and one compared DSC-MRI and ASL^27^. Combinations of modalities applied included DSC-MRI & ASL, DSC-MRI & DCE-MRI, and DSC-MRI & DWI^26,28,29^.

**Figure 2:**
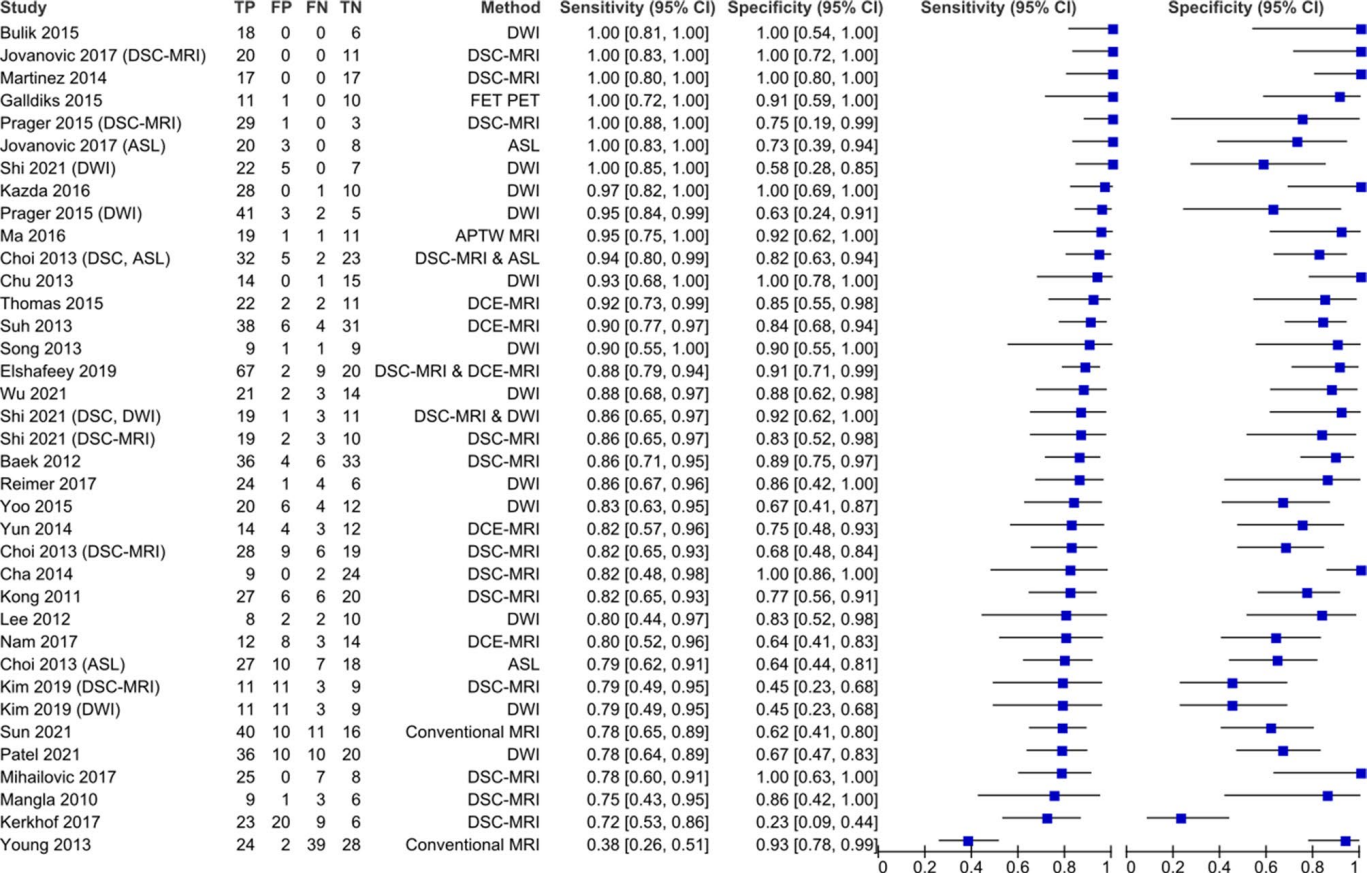
Forest plot assessing various methods of differentiation of pseudoprogression from true progression according to measures of sensitivity and specificity. APTw-MRI = amide proton transfer-weighted MRI; ASL = arterial spin labelling; DCE-pMRI = dynamic contrast-enhanced perfusion MRI; DSC-pMRI = dynamic susceptibility contrast perfusion MRI; DWI = diffusion weighted imaging; FET PET = [18F]fluoroethyltyrosine PET.

All methods were summarised in the non-subgroup analysis (Fig. 2). Three studies reported 100% sensitivity and specificity: two using DSC-MRI, and one using DWI. FET-PET reported high overall sensitivity and specificity in the included study^30^. The included paper that applied APTw-MRI also reported a high diagnostic accuracy (sensitivity = 95%, specificity = 0.92%)^31^. These modalities had insufficient data to be included in the subgroup analysis.

The lowest sensitivity (38%) for identifying tumour progression was reported by Young and colleagues^32^, who examined visual signs such as enhancement on conventional MRI across 93 patients. The only other paper that applied conventional MRI acquisitions used a radiomics-based approach, but still reported relatively low diagnostic accuracy^33^. The lowest specificity for true progression (23%) was reported by Kerkhof and colleagues^34^, which differentiated PsP and TTP by using visual interpretation of relative cerebral blood volume (rCBV) maps from DSC-MRI. Sensitivity tended to be higher than specificity in the majority of included studies.

### Subgroup analysis

**Figure 3:**
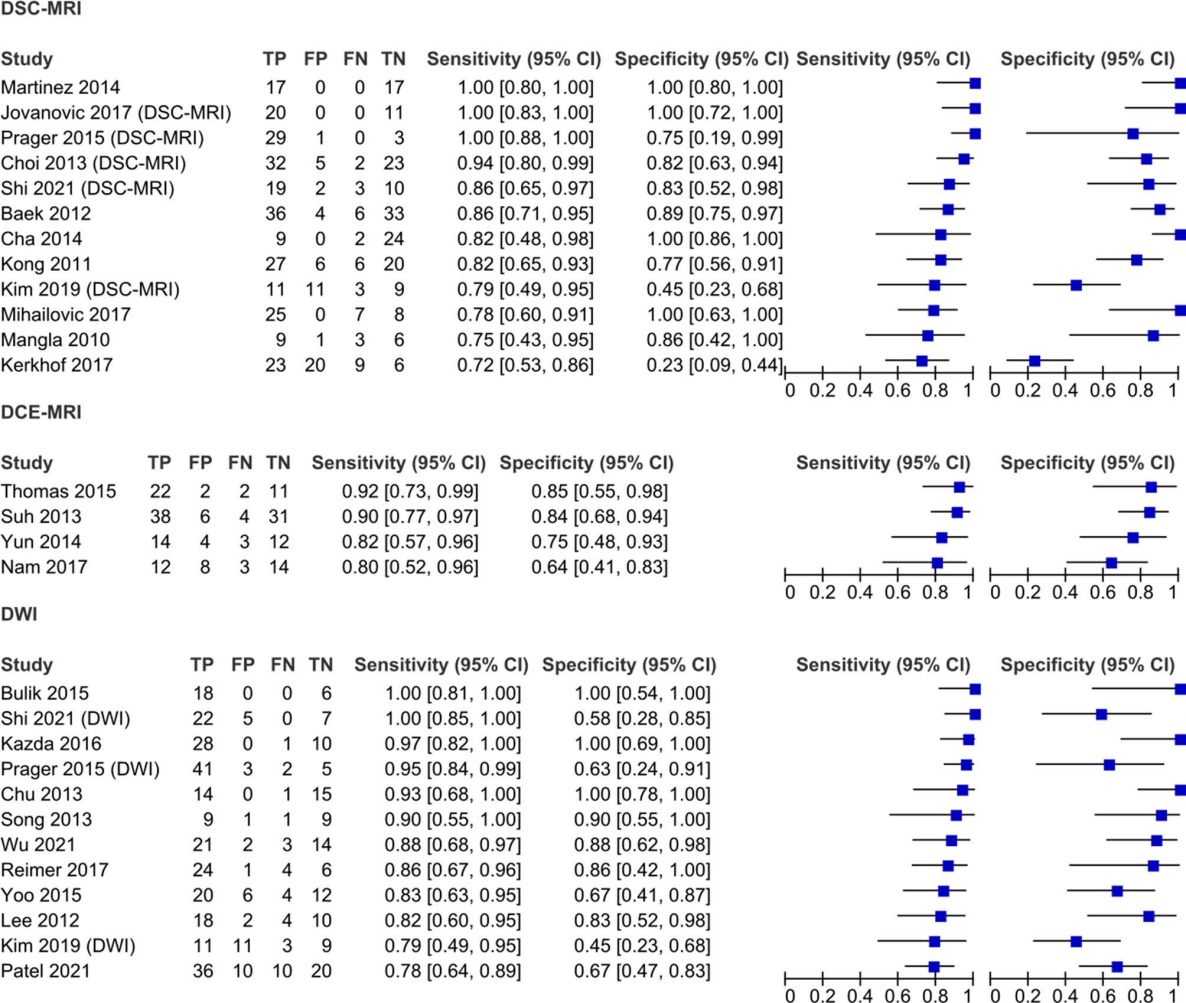
Forest plot subgroup analysis assessing the diagnostic ability of DSC-MRI, DCE-MRI, and DWI. See Fig. 2 for list of abbreviations.

Three distinct methods across 25 studies reported sufficient data to include in a separate set of subgroup analyses: DSC-MRI (n = 12), DCE-MRI (n = 4), and DWI (n = 12). Subgroups included 518, 186, and 459 patients, respectively. Studies by Kim and colleagues^24^, Prager and colleagues^25^, and Shi and colleagues^26^, measured DSC-MRI and DWI separately and thus appear twice in the subgroup forest plot (Fig. 3).

**Figure 4:**
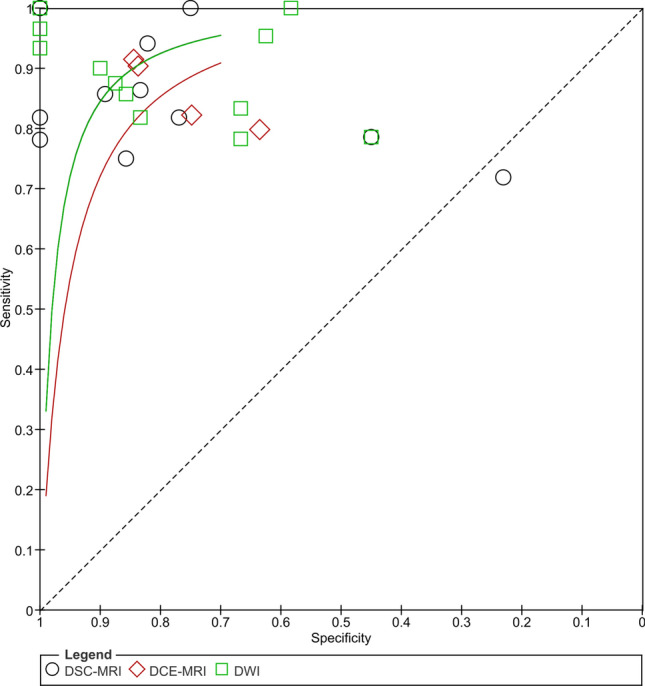
Summary receiver operating characteristics plot comparing diagnostic ability of subgrouped modalities. DSC-MRI, DCE-MRI and DWI are included in the SROC plot and compared according to the mean reported sensitivity and specificity of the studies in the subgroup.

All three subgroups had a high diagnostic accuracy for differentiating PsP from TTP (Figs. 3, 4) supported by high diagnostic odds ratios (Table 2). DWI demonstrated the highest average sensitivity (0.90 [0.84–0.94]), while DSC-MRI demonstrated the highest average specificity (0.88 [0.70–0.96]). A high overall diagnostic accuracy was however demonstrated by all subgroups: DSC-MRI (SROC AUC = 0.93 [0.90–0.95], DOR = 57 [12–268]), DCE-MRI (SROC AUC = 0.90 [0.87–0.93], DOR = 24 [9–60]), and DWI (SROC AUC = 0.93 [0.91–0.95], DOR = 42 [12–268]).

**Table 2:** Summary statistics comparing DSC-MRI, DCE-MRI, and DWI.

	Pooled		Likelihood ratio		DOR	SROC AUC
	Sensitivity	Specificity	Positive	Negative		
DSC-MRI	0.88 [0.81–0.93]	0.88 [0.70–0.96]	7.6 [2.6–22.1]	0.13 [0.07–0.24]	57 [12–268]	0.93 [0.90–0.95]
DCE-MRI	0.88 [0.79–0.93]	0.77 [0.66–0.86]	3.8 [2.4–6.1]	0.16 [0.09–0.29]	24 [9–60]	0.90 [0.87–0.93]
DWI	0.90 [0.84–0.94]	0.82 [0.68–0.91]	5.0 [2.6–9.6]	0.12 [0.07–0.21]	42 [14–126]	0.93 [0.91–0.95]

DOR = diagnostic odds ratio; LR = likelihood ratio; SROC AUC = Summary receiver operator characteristics area under curve.

Heterogeneity for both DCE-MRI and DWI was calculated as *I*^*2*^ = 0%, but high heterogeneity was reported in the DSC-MRI subgroup (*I*^*2*^ = 79%). This heterogeneity was more predominant in the reported specificity (*I*^*2*^ = 85%). The DSC-MRI subgroup had the greatest amount of variation in methodology. However, true heterogeneity is unlikely to be zero in the DCE-MRI and DWI subgroups, and the small sample size may have led to an underestimation^35^.

### Quality assessment

Thirteen of the included 30 studies were determined to have a high risk of bias. Nearly all included studies had a high risk of bias in the index test section. The parameter cut-off values were not pre-specified and instead defined post-hoc. High risk of bias was also apparent in the patient selection category. This was largely due to inclusion of patients who received steroids with standard chemoradiotherapy in some studies. Details of patient enrolment and inclusion/exclusion were also unclear in some studies, and nearly 40% of total included patients presented with PsP, which is higher than previous estimates^8^. There were low applicability concerns observed in the included studies. The full risk of bias table and a more detailed summary of quality assessment across all studies is detailed in Supplementary Material B1 & B2.

## Discussion

The current systematic review and meta-analysis aimed to compare the most promising methods of the differentiation of PsP and TTP in patients with high-grade gliomas. A prior meta-analysis has compared the utility of DWI and PWI (perfusion-weighted imaging) for discriminating PsP and TTP^36^. Consistent with our results, they found the two modalities to be very comparable in terms of diagnostic accuracy. In contrast to the study by Tsakiris and colleagues, our inclusion criteria were not limited to two methods only. Furthermore, we consider each modality in the context of its clinical utility, aiming to provide a recommendation for physicians.

A total of seven PsP and TTP imaging discriminators have been identified in the literature: (1) DSC-MRI, (2) DWI, (3) DCE-MRI, (4) ASL, (5) APTw-MRI, (6) 18F-FET PET, and (7) conventional MRI acquisitions. Three combinations of the methods were identified: (1) DSC-MRI & DCE-MRI, (2) DSC-MRI & DWI, and (3) DSC-MRI & ASL.

The results reported in the current meta-analysis were generally very positive. This may be attributed to a publication bias in which positive results are favoured. Strong positive results on methods for which little data on diagnostic accuracy is available, should be interpreted with caution.

### Diffusion and perfusion-based methods

Our meta-analysis found that DWI, DSC-MRI and DCE-MRI have high potential for differentiating PsP and TTP in patients harbouring HGGs. DSC-MRI and DWI may have some advantage over DCE-MRI, but due to inter-study variations no statistical conclusions can be made. Out of these three methods, DWI demonstrated the highest sensitivity for detecting TTP (0.90 [0.84–0.94]), and DSC-MRI demonstrated the highest specificity (0.88 [0.70–0.96]). Overall accuracy results based on SROC AUC scores were indistinguishable between DWI and DSC-MRI.

Assessment of imaging results using pre-specified parameter cut-off values was associated with higher sensitivity and specificity values in comparison to studies that relied on visual inspection. Kerkhof and colleagues^34^ visually inspected rCBV colour maps to differentiate PsP from TTP, which yielded 72% sensitivity and 23% specificity, both of which were the lowest of the twelve studies included in the DSC-MRI subgroup, which may have negatively skewed averaged results. In contrast, two other included DSC-MRI studies^31,37^ reported 100% sensitivity and 100% specificity using parameter cut-off values to differentiate PsP from TTP. Jovanovic and colleagues used a ratio of 2.89 of normalized CBV between the lesion and normal-appearing tissue, while Martínez-Martínez and colleagues used an rCBV value of 0.9.

Direct comparisons between the diagnostic accuracies of DSC-MRI and DWI were provided in studies by Kim and colleagues^24^, Prager and colleagues^25^, and Shi and colleagues^26^. Kim and colleagues found that the maximum CBV parameter of DSC-MRI and the mean apparent diffusion coefficient (ADC) of DWI differentiated PsP and TTP with the same sensitivity (79%) and specificity (45%) in 34 patients^24^. In the other two studies, DSC-MRI outperformed DWI in specificity, but both reported similarly high sensitivity results^25,26^.

Choi and colleagues^28^ investigated the diagnostic accuracy of DSC-MRI and ASL. The sensitivity and specificity of DSC-MRI were determined as 82.4% and 67.9%, respectively and 79.4% and 64.3%, respectively, for ASL. A combination of the two modalities resulted in an increased sensitivity and specificity of 94.1% and 82.1%, although this did not represent a significant increase in diagnostic accuracy (p = 0.133). Jovanovic and colleagues^27^ separately assessed DSC-MRI and ASL, and quantitative analysis found both methods to yield 100% sensitivity in their patient sample. For specificity, ASL scored 73% compared to 100% for DSC-MRI. All four included diffusion/perfusion-based methods show clinical potential. DSC-MRI is currently the most widely employed, and its protocol and acquisition parameters are already well-defined^38^.

### FET PET

There has been increasing interest in the application of PET in differentiating between PsP and TTP. One study included in this review used 18F-FET PET and found the maximal tumour-to-brain ratio (TBR_max_) differentiates between the two with 100% specificity and 91% sensitivity at a cut-off of 2.3, in a sample of 22 patients^30^. A similar cut-off value of TBR_max_ = 2.55 was reported by Kebir and colleagues^39^. In the same study, a linear discriminant analysis-based algorithm was trained on IDH-wildtype glioblastoma FET PET features and compared the results to a conventional FET PET analysis. The algorithm provided an AUC of 0.93, which was higher than the AUC for TBR_max_ of 0.68.

Several other FET PET studies in the literature were found during the search but did not meet the inclusion criteria, since patients were scanned more than 6 months following diagnosis. This may be a contributing factor to Kim and Shim’s meta-analysis, which found an average sensitivity of 85% and specificity of 88% for detecting PsP using PET, since a later diagnosis predisposes to a more accurate one^40^.

### APTw-MRI

APTw-MRI was used to differentiate PsP and TTP in just one study. Ma and colleagues^31^ found APTw-MRI to correctly identify 19 out of 20 patients in their TTP cohort (95%) and 11 out of 12 patients in their PsP cohort (92%). There was a marked signal increase in the TTP compared to PsP cohort, with an APTWmean cut-off of 2.42% and an APTWmax cut-off of 2.54%. This may be a promising method in the future, but further work on a larger dataset is required.

### Combination methods

Multimodal approaches often demonstrate increased diagnostic accuracy and provide an additional layer of confidence compared to individual modalities. It is reasonable to assume the highest diagnostic accuracy would be achieved from the combination of results from already established modalities. However, the trade-off is the accompanied increase in cost and acquisition time. Regardless, with increasing availability of several above-mentioned modalities, the advantage of combination methods could be considered on a case-by-case basis.

Three combination methods were included in the present review. A combination of *K*^*trans*^ and rCBV maps obtained from DCE-MRI and DSC-MRI acquisitions, respectively, reported high sensitivity (88%) and specificity (91%) in a cohort of 98 patients^29^. The maps could not discriminate between PsP and TTP in the cohort when used individually. Choi and colleagues^28^ combined DSC-MRI with ASL and reported sensitivity and specificity of 94% and 82%, respectively, also finding the combination values higher than the individual methods. Lastly, Shi and colleagues^26^ found that using DSC-MRI and DWI separately produced a specificity of 0.83 and 0.58, respectively. When used in combination, this increased to 92% overall. However, the combination also led to a decrease to 86% in sensitivity overall, despite DWI alone accurately identifying all 22 cases of tumour progression.

### Clinical utility

Despite the large number of studies reporting the diagnostic potential of different imaging protocols, their routine clinical use has not been implemented. A summary of the main clinically relevant parameters is presented in Table 3^41–43^.

**Table 3:** A comparison of each included method.

	Dynamic susceptibility contrast (DSC)	Diffusion-weighted imaging (DWI)	Dynamic contrast enhanced (DCE)	Arterial spin labelling (ASL)	Amide proton transfer-weighted (APTw)	18F-FET PET
Sensitivity/specificity*	0.88 / 0.88	0.90 / 0.82	0.88 / 0.77	-	-	-
Use of contrast agent/radioactive tracer	✓	✖	✓	✖	✖	✓
Acquisition time	~ 1–2 min	~ 1.5–3 min	~ 5–10 min	~ 3–5 min	~ 5–10 min	~ 30–50 min
Signal-to-noise ratio	Low	Low	High	Low	Low	High
Parameters	CBV, CBF, MTT	ADC	*k*^*trans*^*, v*_*p*_*, v*_*e*_, IAUC	CBF	Signal intensity (max, min, mean and range)	Tumour-to-brain uptake ratio (TBR)

* Based on computed subgroup averages.

An inherent limitation of using perfusion-weighted imaging is that while perfusion parameters are generally lower in PsP, the associated inflammatory response is likely to influence perfusion and lead to increased perfusion parameters such as rCBV^44^. Similar effects have been seen with DWI as a result of radiation necrosis, suggesting decreased ADC may not always reflect a high cellularity and TTP^45^. However, both PWI and DWI appear to demonstrate high overall diagnostic accuracy.

As the most commonly used perfusion MRI modality, DSC-MRI may be preferable for standard protocol due to its high clinical availability and short acquisition time that can be under one minute^43^. The standardisation of rCBV discriminating cut-off values is limited by numerous potential imaging and data processing artifacts impeding accurate perfusion quantification as outlined by Willats and Calamante’s 39 steps for accurate perfusion of DSC-MRI data^46^. One of the most widely discussed issues is the possibility of contrast agent leakage into extracellular tissue, known as T1 shine-through effect^47^. Application of model-based leakage corrections is advised for single-echo gadolinium-based DSC-MRI to account for the extent of vascular permeability^48^.

DCE-MRI has a high signal-to-noise ratio compared to the other MR-perfusion techniques^49^. The main limitation of this method is the relatively long data acquisition time, often over several minutes^50^. Similar to other perfusion techniques, full quantification remains challenging due to difficulties with DCE tracer modelling. Efforts are currently undertaken to resolve issues related to accurate quantification of perfusion techniques. The establishment of taskforces such as the Quantitative Imaging Biomarkers alliance will facilitate clinical implementation of methods by providing reference measures and guidelines for best practices^51^.

ASL was a less frequently reported discriminating method compared to other perfusion methods. The main advantage of ASL over DSC-MRI is that it does not require a gadolinium-based bolus injection. It may therefore be more suitable for patients with contraindications to administration of contrast agents^52^. Furthermore, ASL can acquire entirely quantitative values of cerebral blood flow (CBF). A non-significant increase in sensitivity and specificity was observed when CBF measures acquired using ASL was combined with DSC-MRI, compared to use of the methods^28^. Jovanovic and colleagues^27^ concluded that the diagnostic accuracy of ASL was sufficient to replace DSC-MRI and therefore, avoid repeat follow-up contrast injections. An important consideration of ASL is the longer acquisition time of 8–10 min at 1.5 T and 4–5 min at 3 T as well as significantly lower signal-to-noise ratio (SNR) compared to other perfusion methods^43^.

APTw is a novel imaging technique demonstrated to detect the increased mobile protein content in brain tumours^53^. Its full potential is yet to be established as U.S. Food and Drug Administration (FDA) approval of 3D-APTw for use on 3 T clinical MRI scanners was granted in 2018^54^. However, APTw examinations may be time consuming (~ 5–10 min) and are susceptible to magnetic field inhomogeneities^55^. Some work aims to optimise the signal-to-noise ratio and image acquisition speed^56^. APTw is a promising method with initial studies reporting a high diagnostic accuracy, but larger datasets are needed to compare its performance against other techniques.

Despite the high reported sensitivity and specificity of 18F-FET PET, a long acquisition time of 50 min as reported by Galldiks and colleagues^30^ limits clinical potential. Since 18F-FET PET relies on administration of labelled amino acid analogue, patients in the study were also required to fast for at least 12 h before scanning. In contrast to other radiotracers, the half-life of fluorine-18 is long enough to allow for off-site production. The requirement for pharmacokinetic analysis with compartment modelling^57^ further limits potential for clinical implementation.

### Future directions

Quantitative methods offer a more objective approach towards finding patterns in clinical data and enable more accurate diagnosis compared to qualitative methods^58,59^. Jang and colleagues^60^ recently applied a deep learning approach using convolutional neural networks to the differentiation of pseudoprogression and true progression and achieved a sensitivity of 87% and a specificity of 94.5%. Another study found a benefit of the combination of hypervascularity, cellularity and permeability parameters over single parameter measurements to distinguish the conditions^61^. The need for large datasets for training and testing radiomics models has led to a general lack of power, therefore future research should focus on increasing accessibility and data availability. National support for the scaling of technology and the potential use of artificial intelligence to aid clinical decision making has been outlined in the NHS Long Term Plan^62^.

## Conclusion

Our systematic review and meta-analysis found DWI and DSC-MRI to have the highest diagnostic accuracy for differentiating between PsP and TTP. Considering the acquisition time and availability, DSC-MRI holds high potential for clinical implementation. The risk of repeat contrast agent injections required for DSC-MRI could be offset with the substitution of DSC-MRI for ASL. There was a clear advantage of using parameter cut-offs, over methods that relied on qualitative visual inspection. The diagnostic accuracy of methods such as PET, APTw-MRI, clinically feasible combination methods, and quantitative multiparametric techniques should be investigated in large-scale studies.

## Supplementary Information

Supplementary Information.
