# Supplementary material for: Discriminators of pseudoprogression and true progression in high-grade gliomas: A systematic review and meta-analysis
Source: Sci Rep. 2022 Aug 2;12:13258. doi: 10.1038/s41598-022-16726-x (PMC9345984; doi:10.1038/s41598-022-16726-x)
Supplement: Supplementary file 1 — Supplementary Information. [file 41598_2022_16726_MOESM1_ESM.pdf]

Discriminators of pseudoprogression and true progression in high-grade gliomas: A systematic review and meta-analysis

Supplementary Material

Chris Taylor BSc[1*], Justyna O. Ekert MPhil[2], Viktoria Sefcikova MSc[1], Naomi Fersht MB BChir PhD MRCP FRCR[3], George Samandouras MD, FRCS[1,4]

1. UCL Queen Square Institute of Neurology, University College London, Queen Square, London, United Kingdom
2. Wellcome Centre for Human Neuroimaging, University College London, 12 Queen Square, London, United Kingdom
3. Department of Oncology, University College London Hospitals NHS Foundation Trust, London, United Kingdom.
4. Victor Horsley Department of Neurosurgery, The National Hospital for Neurology and Neurosurgery, Queen Square, London, United Kingdom

*Corresponding Author:

Chris Taylor
UCL Queen Square Institute of Neurology
Gower St, Bloomsbury, London
WC1E 6BT
skgtayl@ucl.ac.uk
07523016269
0000-0002-3289-5860

Supplementary Material A

Search Strategy

Embase, Medline, Web of Science and Google Scholar were searched separately on the 20th May 2022 with specific search criteria.

- Embase: 1974 to April 27 2022
- Ovid MEDLINE(R) All 1946 to April 27 2022
- Web of Science Core Collection
- Google Scholar (first 200 results)

Prior to searching, the protocol was registered on PROSPERO with ID “CRD42022218217”.

EMBASE (1062 results)

1. exp glioma/ or glioma$.mp. or exp oligodendroglioma/ or oligodendroglioma$.mp. or exp ependymoma/ or ependymoma$.mp. or exp gliosarcoma/ or gliosarcoma.mp. or exp astrocytoma/ or astrocytoma$.mp. or exp glioblastoma/ or glioblastoma$.mp. or GBM.mp. or brain tumo?r$.mp. or brain glioma$.mp. or glia? tumo?r$.mp. or low grade glioma$.mp. or high grade glioma$.mp. or LGG.mp. or HGG.mp.
2. (pseudoprogression or pseudo-progression or psPD or PsP).mp.
3. 1 and 2

MEDLINE (448 results)

1. exp glioma/ or glioma$.ti,ab. or exp oligodendroglioma/ or oligodendroglioma$.ti,ab. or exp ependymoma/ or ependymoma$.ti,ab. or exp gliosarcoma/ or gliosarcoma.ti,ab. or exp astrocytoma/ or astrocytoma$.ti,ab. or exp glioblastoma/ or glioblastoma$.ti,ab. or GBM.ti,ab. or brain tumo?r$.ti,ab. or brain glioma$.ti,ab. or glia? tumo?r$.ti,ab. or low grade glioma$.ti,ab or low-grade-glioma$.ti,ab or high grade glioma$.ti,ab. or high-grade-glioma$.ti,ab. or LGG.ti,ab. or HGG.ti,ab.
2. (pseudoprogression or pseudo-progression or psPD or PsP).ti,ab.
3. 1 and 2

Web of Science (908 results)

1. TS = (“glioma*” OR “oligodendroglioma*” OR “ependymoma*” OR “gliosarcoma*” OR “astrocytoma*” OR “glioblastoma*” OR “GBM” OR “brain tumo$r*” OR “glial tumo$r” OR “low grade glioma*” OR” low-grade-glioma*” OR “high grade glioma*” OR “high-grade-glioma*” OR “LGG” OR “HGG”)
2. TS = (“pseudoprogression” OR “pseudo-progression” OR “psPD” OR “PsP”)
3. 1 and 2

Google Scholar (First 200 results)

(oligodendroglioma | astrocytoma | glioblastoma | GBM | glioma | brain tumour | brain tumor | LGG | HGG | low grade glioma | high grade glioma | glial tumor | glial tumour | glia tumor | glia tumour) (pseudoprogression | pseudo-progression | psPD | PsP)

Supplementary Material B1

Risk of bias and applicability table assessing the quality of included studies according to the Quality Assessment of Diagnostic Accuracy Studies-2 (QUADAS-2) tool. Martinez et al., Kerkhof et al., Kong et al., Mangla 2010 et al., Song et al., and Thomas et al., were all found to include at least one patient that had received steroid treatment.

Risk of bias domains

Study	D1	D2	D3	D4	Overall
Baek 2012	X	X	+	-	X
Bulik 2015	X	X	+	-	X
Cha 2014	+	X	+	X	-
Choi 2013	X	X	+	-	-
Chu 2013	-	X	+	+	-
Elshafeey 2019	-	X	+	X	X
Galldiks 2015	X	X	+	-	X
Jovanovic 2017	+	X	-	+	+
Kazda 2016	X	X	-	X	X
Kerkhof 2017	X	-	+	+	-
Kim 2019	+	X	+	+	+
Kong 2011	X	X	+	-	X
Lee 2012	-	X	+	+	-
Ma 2016	-	X	+	-	-
Mangla 2010	X	X	X	+	X
Martinez 2014	X	X	-	-	X
Mihailovic 2017	+	X	+	+	+
Nam 2017	X	X	+	+	-
Patel 2021	+	X	+	+	-
Prager 2015	+	X	+	+	+
Reimer 2015	+	X	X	-	X
Shi 2021	X	X	+	+	X
Song 2013	X	X	+	+	X
Suh 2013	+	X	+	+	+
Sun YZ 2021	+	X	-	+	-
Thomas 2015	X	X	X	-	X
Wu XF 2021	X	X	-	-	X
Yoo 2015	-	+	+	+	+
Young 2011	X	X	+	+	-
Yun 2014	-	X	+	+	-

Domains:
D1: Patient selection.
D2: Index test.
D3: Reference standard.
D4: Flow & timing.

Judgement
X High
- Some concerns
+ Low

Supplementary Material B2

Summary of risk of bias across all studies according to the risk of bias table in Figure 5 assessing the quality of included studies according to the Quality Assessment of Diagnostic Accuracy Studies-2 (QUADAS-2) tool.

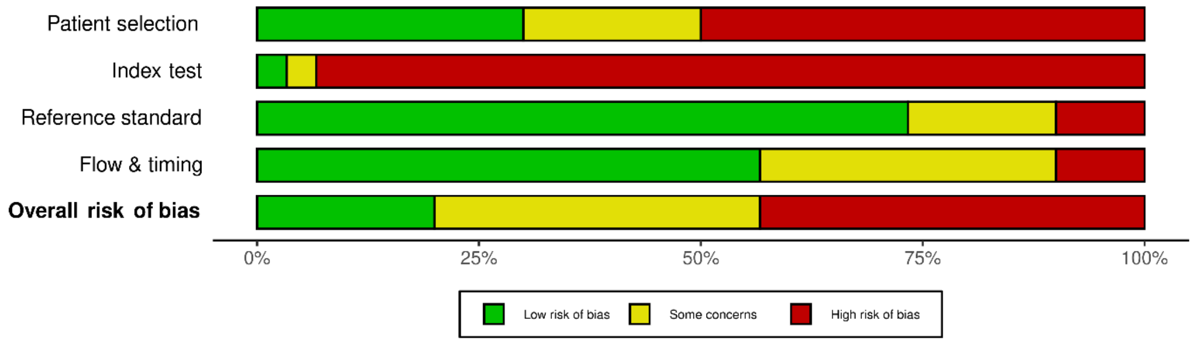

Supplementary Material C

This review assessed studies including sufficient data for the following parameters:

(1) Adult glioma patients (≥16 years old) that received radiotherapy following a prior diagnosis

(2) Studies assessed a method of differentiation of pseudoprogression from true progression via measures of sensitivity and specificity

(3) Reference standard for identification of PsP was the use of the RANO criteria and/or expert assessment. Patients that died within 6 months of radiotherapy were not classified as pseudoprogression

(4) Record of the proportion of the population which are confirmed to exhibit pseudoprogression

(5) Confirmation of pseudoprogression must not exceed 6 months following radiotherapy, since presentation after 6 months is clinically defined as radiation necrosis and not pseudoprogression
